# The Protective Effect of Lycium Ruthenicum Murr Anthocyanins in Cr (VI)-Induced Mitophagy in DF-1 Cells

**DOI:** 10.3390/life12081115

**Published:** 2022-07-25

**Authors:** Shuhua Guo, Mengzhu Qi, Hongyan Li, Yukun Cui, Changxi Qi, Guodong Cheng, Meiyun Lv, Pimiao Zheng, Jianzhu Liu

**Affiliations:** 1 College of Veterinary Medicine, Shandong Agricultural University, Tai’an 271018, China; shguo999@163.com (S.G.); 18764880103@163.com (G.C.); lvmeiyun2022@163.com (M.L.); 2 Research Center for Animal Disease Control Engineering, Shandong Agricultural University, Tai’an 271018, China; 15588042903@163.com (M.Q.); cuiyukunnn@163.com (Y.C.); 15589985049@163.com (C.Q.); 3 Central Hospital of Tai’an City, Tai’an 271018, China; 13561785027@163.com

**Keywords:** anthocyanin, chromium (VI), DF-1 Cells, mitophagy, PINK1/Parkin

## Abstract

Cr (VI) is an extremely toxic environment and professional pollutant that seriously damages mitochondrial dysfunction when it enters a cell. Anthocyanins possess anti-oxidant, antiaging, and antifatigue properties. The regulatory effect of Lycium ruthenicum Murr anthocyanin (LRMA) on Cr (VI)-induced mitophagy in DF-1 cells was determined. The experimental design was divided into blank group, groups subjected to Cr (VI) and Cr (VI), and LRMA co-treatment groups. Cell viability was determined by the CCK-8 assay. Mitochondrial membrane potential (MMP) and reactive oxygen species (ROS) were assessed by flow cytometry and immunofluorescence. Mitophagy was monitored by ELISA and Western blot. Data showed that Cr (VI) caused the overexpression of autophagy-related proteins (LC3, Beclin-1) and reduced the expressions of autophagy protein p62 and TOMM20. Compared with the Cr (VI) group, the LRMA group showed considerably decreased mitochondrial damage and mitophagy. LRMA decreased the mitochondrial protein expression of PINK1 and Parkin’s transfer from the cytoplasm to mitochondria. LRMA may confer protective effects by reducing PINK1/Parkin-mediated mitophagy in Cr (VI)-induced DF-1 cell models.

## 1. Introduction

The discovery of chromium (Cr) can be traced back to 1797, when this new type of mineral was found by French chemist Louis N. Vauquelin in red lead ore, which he reduced with the chemical element carbon to obtain Cr. However, Cr from the zero valence state to the highest valence state (hexavalent state) is always stable as Cr (VI) and Cr (III) [1,2,3]. Cr (VI) enters the biological body. It easily penetrates the outer cell membrane, enters the cells, and exerts a strong oxidizing effect, thereby threatening the normal liver metabolism of the cells and posing a significant threat to the body. Large organisms exposed to a Cr (VI) environment could show liver damage, skin and mucosal ulcers, kidney damage, gastric mucosal damage, and internal organ bleeding. Many in vivo and in vitro experiments have shown that the cytotoxicity caused by Cr (VI) compounds could be passed on to the next generation, leading to base mutations, DNA damage, and genetic variation [4]. Cr (VI) easily accumulates in organisms and has strong virulence and migration ability. Cr (VI) poses a great threat to human health development and animal survival and has attracted the attention of domestic and foreign researchers. Heavy metal pollution has always been the focus of global attention.

Anthocyanin belongs to the flavonoid family of phenolic compounds [5]; these are mainly extracted from various fruits, petals, and colored vegetables [6]. The beneficial effects of anthocyanin on the treatment of some conditions and diseases, such as nervous system disease, cardiovascular disease, and inflammation, as well as cancer [7], have received considerable attention [8]. These diseases are mainly manifested as oxidative stress in the cells, perhaps as a result of the imbalance in the production of reactive oxygen or reactive nitrogen with the anti-oxidant defense system in vivo [9,10]. These reactions could attack biomolecules, such as lipids, DNA, and proteins [11], causing tissue damage and triggering cell death pathways. The overproduction of reactive oxygen radicals could be a typical representation of oxidative stress. Anthocyanins have a strong ability to eliminate free radicals. With an increasing concentration, the scavenging ability of free radicals was significantly improved [12], similar to other anti-oxidants [13]. Anthocyanins could also achieve anti-cancer and anti-tumor effects by regulating related signal pathways, promoting cancer cell apoptosis and autophagy, and enhancing chemotherapy sensitivity.

Mitochondria are essential organelles that provide cells with energy and metabolic intermediates and play a crucial role in defending against toxic stimuli and maintaining homeostasis [14,15]. Mitochondria is also the key place where ROS is produced [16]; they play a central role in the onset and progression of the disease. When mitochondria undergo oxidative stress, dysfunction occurs, leading to various diseases [17]. Many studies have reported the effective mitochondrial damage treatment for Parkin disease [18,19]. Mitochondrial protection plays an essential role in the defense network under environmental stress conditions. Many recent studies have shown that mitophagy is the primary mechanism underlying mitochondrial quality control [20,21]. 

Mitophagy controls mitochondrial quality by selectively removing damaged or excess mitochondria [22,23]. Multiple signal access areas through which mitophagy could take place have been postulated, e.g., PINK1/Parkin [24]. The activation of the outer mitochondrial membrane protein by PINK1/Parkin signal further triggers the transport of the ubiquitin protein receptor SQSTM1/p62 to the mitochondria [25,26]; it binds to the autophagy substrate protein LC3 and finally undergoes lysosome degradation. SQSTM1/p62 and LC3 are critical proteins in mitophagy. Protein ATG5 and Beclin-1 are a family of autophagy-related proteins and are among the best-characterized autophagy-related proteins. When the toxin enters the cells, it regulates cell growth progression through the immune system. Mitophagy is changed by a complex signal network, including various regulatory factors, rather than a simple linear regulatory pathway. Narendra and colleagues found that, when autophagy occurs, Parkin, an E3 ubiquitin ligase, is transferred from the cytoplasm to the mitochondria [27,28].

Our research team has shown that Cr (VI) could induce intracellular mitophagy in DF-1 cells [29]. Anthocyanin is a natural anti-oxidant; some researchers suggested that anthocyanin plays a vital role in the development of numerous aging diseases via various anti-oxidant pathways [30]. Thus, using anthocyanins may be an effective strategy for addressing various oxidative stresses. This study was conducted to explore whether the anti-oxidant capacity of anthocyanins could alleviate mitochondrial damage and mitophagy in DF-1 cells caused by Cr (VI) and provide a heavy theoretical basis for seeking an antidote to Cr (VI) poisoning.

## 2. Materials and Methods

### 2.1. Experimental Materials

K_2_Cr_2_O_7_ was acquired from Kaitong Chemical (Shanghai, China). Total LRMA was a gift from Professor Ding Xinhua, School of Plant Protection, Shandong Agricultural University. DMEM/high glucose and Trypsin were purchased from Gibco Company (Grand Island, NE, USA). Fetal bovine serum was purchased from Biological Industries (State of Israel). MMP detection kit, ROS (DCFH-DA), and TOMM20 antibody were supplied from the Beyotime Institute of Biotechnology (Haimen, Jiangsu, China). LC3 antibody was purchased from Abcam. p62, Beclin-1, ATG5, and GADPH antibodies were obtained from Proteintech (Chicago, IL, USA). Parkin antibody (A0968) was purchased from ABclonal (Wuhan, China). Chicken PINK1 ELISA kits were purchased from Enzyme-linked Biotechnology (Shanghai, China). BCA kit was bought from ComWin Biotech (Beijing, China).

### 2.2. Cell Treatment

Cr (VI) at 40 μM and different concentrations of LRMA were used as single and co-treatment components, and then the cells were cultured at 6 h. The cells were divided into four groups: (1) blank group; (2) 40 μM Cr (VI); (3) 40 μM Cr (VI) + 50 μg/mL LRMA; and (4) 40 μM Cr (VI) + 100 μg/mL LRMA.

### 2.3. Cell Viability Assay

The cell viability of the DF-1 cells was measured with a CCK-8 assay kit (Monmouth Junction, NJ, USA), following the manufacturer’s instructions. Cells were grown in 96-well plates until monolayers were formed, after which CCK-8 reagent was added to each well. Following incubation for 2 h at 37 °C, the absorbance at 450 nm was measured using a microplate reader, and the viability of the DF-1 cells was expressed as the blank group’s percentage.

### 2.4. ELISA Assay

Total cellular proteins were obtained after DF-1 cells were treated with Cr (VI) and LRMA. Then, the contents of mitophagy-related protein (PINK1) and anti-oxidants (ROS) were determined by ELISA. The DCFH probe was passed through the cell membrane, and the probe was transferred into the cells. Detecting the fluorescence of DCFH could determine the level of reactive oxygen species in the cells. The ELISA assay was measured according to the operating instructions. Following the treatment, the cells were incubated with DCFH-DA for 20 min at 37 °C and then the fluorescence content was measured using flow cytometry.

### 2.5. Measurement of MMP

Mitochondrial membrane potential (MMP) was observed using the JC-1 kit, which was purchased from the Beyotime Biotechnology Company. DF-1 cells were incubated with the JC-1 probe at 37 °C for 30 min. Then, DF-1 cells were washed thrice with PBS to eliminate the JC-1 probe. Subsequently, the change in MMP was measured by flow cytometry.

### 2.6. Western Blot Analysis

The total proteins of cells or mitochondrial proteins were collected using RIPA lysis buffer (Beyotime, Beijing, China) on ice. The cytoplasmic and mitochondrial proteins were gathered using a Mitochondria Isolation Kit (Kaiji Biotechnology Co., Ltd., Jiangsu, China). Protein concentration was determined by BCA Protein Assay Kit (ComWin Biotech Co., Ltd., Beijing, China). Protein samples (12–15 μg) were electrophoresed on 10–12% gradient SDS-PAGE gel. Then, electrophoretically separated proteins were transferred to PVDF membranes. The membranes were closed and incubated with antibodies against LC3II, p62, ATG5, Beclin-1, Parkin, GAPDH, and TOMM20 at 4 °C overnight, followed by goat anti-mouse IgG and goat anti-rabbit IgG antibodies. The membrane was visualized using chemiluminescent fluid and ImageJ software for quantitative analysis.

### 2.7. Statistical Analysis

Statistical processing was determined using IBM SPSS Statistics 26. All results in the present study were analyzed by one-way ANOVA and by Tukey’s comparison test. When *p* < 0.05, it was determined to have a statistically significant difference.

## 3. Results

### 3.1. Effect of LRMA Cells’ Viability

**Figure 1:**
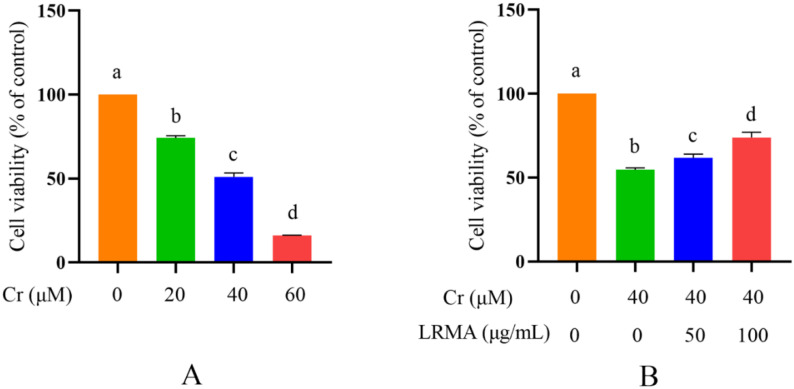
Cr (VI) induces cell death in DF-1 cells. LRMA decreases cell death in Cr (VI)-induced cells. (**A**,**B**) Cellular viability was detected by CCK-8 assay. Different doses of LRMA and Cr (VI) were added to the medium of DF-1 cells for 6 h. Data were presented as mean ± SD (*n* = 3). Means that do not share common letters (a–d) differ significantly (*p* < 0.05).

To establish the poisoning model of Cr (VI) and the treatment model of LRMA in the DF-1 cells in vitro, previous research pointed out the effect of Cr (VI) on the occurrence of mitophagy in DF-1 cells. DF-1 cells were treated with different doses of Cr (VI) for approximately 6 h [29]. Unlike this experiment, the CCK-8 assay can be used to detect cells’ viability. The results obtained are consistent with the previous results (Figure 1A). Then, Figure 1B cites the concentration of anthocyanins used for Cr (VI)-induced autophagy in the previous research. The types of cells and anthocyanins studied are different. Therefore, the use of CCK-8 assay was remedied with different doses of LRMA for 6 h in DF-1 cells. Compared with the blank group, LRMA dose-dependently increased the viability of DF-1 cells (Figure 1B). Accordingly, LRMA at 50 and 100 μg/mL was used in subsequent studies. These data showed that Cr (VI) alleviated toxicity in DF-1 cells when administered with a certain dose of LRMA.

### 3.2. Effect of LRMA on ROS Accumulation

**Figure 2:**
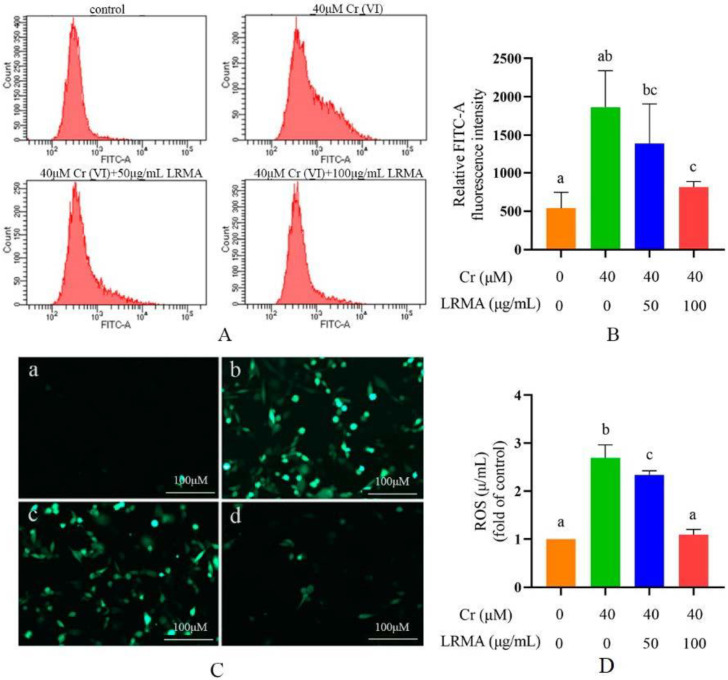
LRMA treated anti-oxidant damage in Cr (VI)-induced cells. (**A**,**B**) Flow cytometry with DCFH-DA dyeing was used to observe the ROS of DF-1 cells. (**C**) ROS production was used to observe the green fluorescence of DF-1 cells. (a: control, b: 40 μM Cr (VI), c: 40 μM Cr (VI) + 50 μg/mL LRMA, d: 40 μM Cr (VI) + 100 μg/mL LRMA). (**D**) The content of anti-oxidants was determined by ELISA assay. Different doses of LRMA and Cr (VI) were added to the medium of DF-1 cells for 6 h. Data were presented as mean ± SD (*n* = 3). Means that do not share common letters (a–c) differ significantly (*p* < 0.05).

The DCFH-DA probe staining was used to observe the cellular oxidative stress on DF-1 cells, according to the fluorescence intensity and flow cytometry method for qualitative and quantitative analysis. Compared with the blank group, Cr (VI) increased the intensity of green fluorescence (Figure 2C). Cr (VI) treatment significantly promoted the generation of ROS, indicating that oxidative stress occurred in DF-1 cells (Figure 2A,B). Subsequently, we also determined whether LRMA exerts anti-oxidant effects to relieve oxidative stress. Therefore, DF-1 cells were incubated in LRMA and Cr (VI) for 6 h. A data assay was performed to assess the ROS of LRMA-treated cells. Compared with the blank group, LRMA treatment reduced the number of green fluorescence (Figure 2A,B). LRMA repressed the oxidative stress of DF-1 cells, which reduced cell damage.

### 3.3. Effect of LRMA on MMP

**Figure 3:**
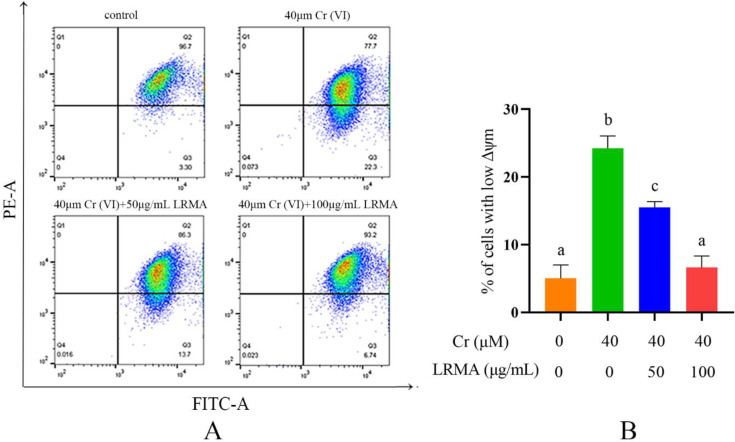
LRMA treated mitochondria damage in Cr (VI)-induced cells. (**A**,**B**) Flow cytometry with JC-1 dyeing was used to observe the MMP of DF-1 cells. Different doses of LRMA and Cr (VI) were added to the medium of DF-1 cells for 6 h. Data were presented as mean ± SD (*n* = 3). Means that do not share common letters (a–c) differ significantly (*p* < 0.05).

To further determine the mitochondrial damage on DF-1 cells, we monitored the expression of MMP and measured MMP by flow cytometry assay. First, the MMP was monitored using JC-1 staining under Cr (VI) stimulus. As shown in Figure 3A,B, mitochondrial potential decreased in Cr (VI)-treated cells compared with the blank group, indicating a decrease in MMP. Therefore, LRMA and Cr (VI) were incubated on DF-1 cells for 6 h. Flow cytometry was performed to evaluate the MMP of LRMA-treated cells. Compared with the blank group, LRMA treatment increased the MMP (Figure 3A,B). These data indicated that LRMA alleviated the damage of MMP in DF-1 cells, which in turn reduced cell damage.

### 3.4. LRMA Inhibited Cr (VI)-Induced Autophagy

**Figure 4:**
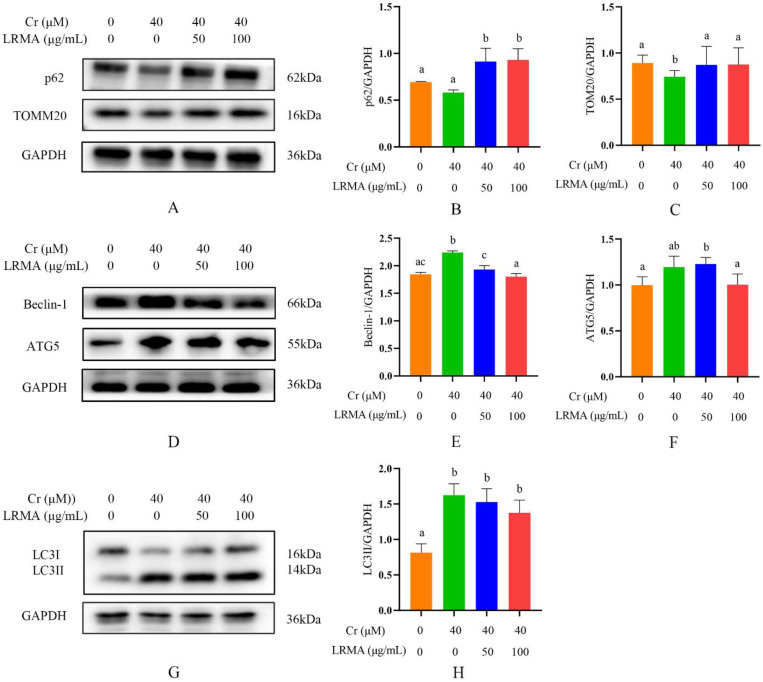
Effects of LRMA on Cr (VI)-induced p62, TOMM20, ATG5, Beclin-1, and LC3II protein expression. (**A**,**D**,**G**) Whole cells lysate was gathered for Western blot analysis for p62, TOMM20, ATG5, Beclin-1, LC3II, and GAPDH. (**B**,**C**,**E**,**F**,**H**) Optical densitometry analysis of protein signals on Western blots. The analysis results were divided by GAPDH value of the same protein samples to determine the proportion of protein expression. Different doses of LRMAand Cr (VI) were added to the medium of DF-1 cells for 6 h. Data were presented as mean ± SD (*n* = 3). Means that do not share common letters (a–c) differ significantly (*p* < 0.05).

p62 is a selective autophagy-related receptor that plays a vital bridge role between ubiquitinated proteins and autophagosomes in cells. LC3 activation is the molecular characteristic of autophagy cells. TOMM20 is a membrane protein in the mitochondria. Beclin-1 is a homologous gene of yeast Atg6. ATG5 is an essential protein involved in the extension of autophagy phagocytic cells during autophagy. Western blot analysis was conducted to evaluate autophagy-related protein in DF-1 cells. The expressions of autophagy-related proteins were monitored to determine Cr (VI) on DF-1 cells. As shown in Figure 4G,H, compared with the blank group, LRMA treatment downregulated LC3II, ATG5, and Beclin-1 expressions and decreased the LC3II (Figure 4D–H). Moreover, p62 and mitochondrial TOMM20 expressions were also increased after adding LRMA (Figure 4A–C). Autophagy-related protein markers, such as Beclin-1 and ATG5, were also decreased in LRMA-treated DF-1 cells compared with the Cr (VI) group. Together, these data showed that LRMA suppressed autophagy activity in DF-1 cells.

### 3.5. LRMA Inhibited Cr (VI)-Induced Mitochondria Autophagy

**Figure 5:**
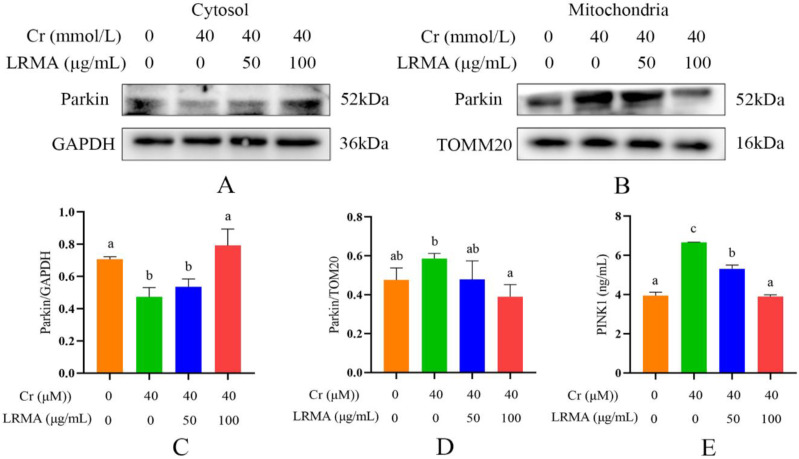
Effects of LRMA on Cr (VI)-induced Parkin and PINK1 protein expression. (**A**,**B**) Whole cell lysate was gathered for Western blot analysis for Parkin, TOMM20, and GAPDH. (**C**,**D**) Optical densitometry analysis of Parkin protein signals on Western blots; the Parkin analysis result was divided by the GAPDH value in the cytoplasm of the same protein sample and the TOMM20 value on the mitochondria to determine the proportion of protein expression. (**E**) The concentration of PINK1 was measured by ELISA assay. Different doses of LRMA and Cr (VI) were added to the medium of DF-1 cells for 6 h. Data were presented as mean ± SD (*n* = 3). Means that do not share common letters (a–c) differ significantly (*p* < 0.05).

Previous studies have shown that mitophagy was mainly dominated by the PINK1/Parkin pathway [31]. Our data demonstrated that autophagy factors, including Parkin and PINK1, were downregulated by Cr (VI) (Figure 5A). These data indicated that Cr (VI) controlled mitophagy by activating the PINK1/Parkin pathway. Therefore, we guessed whether the PINK1/Parkin pathway is involved in LRMA-related mitophagy. Western blot and ELISA indicated the decrease in the transfer of Parkin from the cytoplasm into the outer mitochondrial membrane and the reduction in PINK1 accumulation in the outer mitochondrial membrane (Figure 5A–E). The above data showed the inhibitory action of LRMA on mitophagy. Therefore, LRMA could alleviate the occurrence of mitophagy in DF-1 cells induced by Cr (VI).

## 4. Discussion

In recent years, mitophagy mediator E3 ubiquitin-protein ligase PARK2/Parkin has been studied extensively by scholars [24]. Since the discovery of mitophagy, it has been confirmed that effectors, such as the PINK1/Parkin pathway, Bnip3, NIX, and FUNDC1, are involved in mammalian mitophagy. The PINK1/Parkin pathway has become an essential pathway regulating mitophagy and mitochondrial function [32]. Studies have explored the effects of the PINK1/Parkin pathway on doxorubicin (DOX)-induced mitochondrial damage and cardiotoxicity. Cytotoxicity and mitochondrial toxicity depend on the concentration of doxorubicin, including accumulation of mitochondrial superoxide and depolarization of MMP [33]. Mitochondrial DNA copy number and ultrastructure changes and doxorubicin induce the increase in the expressions of mitochondrial autophagosome LC3 and Beclin1, decrease in the expression of p62, and increase in the co-localization of LC3 in mitochondria. Recent studies have shown that PINK1/Parkin is activated to depolarize mitochondria and trigger mitochondrial autophagy. p62/SQSTM1 is the ubiquitin-binding protein associated with protein aggregates and damaged mitochondria [34]. Optineurin/OPTN is another ubiquitin-binding protein related to autophagy and impaired mitochondrial depolarization [35]. Both p62/SQSTM1 and OPTN are recruited to ubiquitinate substrates, combine with autophagosome LC3, and induce degradation [36].

**Figure 6:**
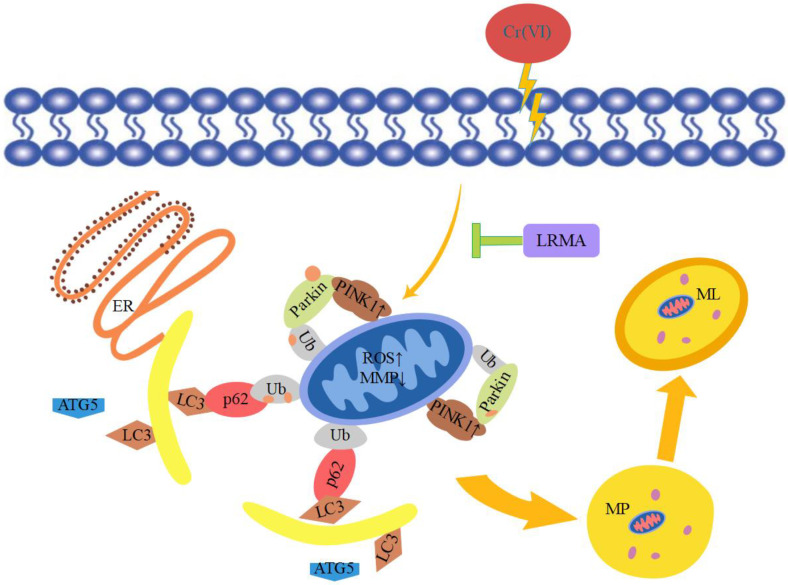
Graphical indication of the mechanism of LRMA suppression Cr (VI)-induced mitophagy injury. PINK1/Parkin-mediated mitophagy involves the recognization of damaged mitochondria by PINK1 and activation of Parkin. This leads to ubiquitination of outer mitochondrial membrane-associated proteins, which are then recognized by autophagic receptors such as p62. This receptor can bridge the cargos with LC3 to activate mitophagy.

Cr (VI) could cause oxidative stress in cells, which results in mitochondria damage and mitophagy. Cr (VI) induces the process of mitophagy by affecting mitochondrial damage and by altering autophagy-related protein levels in PINK1/Parkin signaling. The beneficial effects of anthocyanins against various diseases induced by various oxidative stresses have received much attention. Our previous studies have suggested that anthocyanin may alleviate mitochondrial dysfunction by alleviating endoplasmic reticulum stress in Cr (VI)-induced DF-1 cell models [37]. Recent evidence shows the involvement of anthocyanin in the pathogenesis of various disorders and as an important role of anti-oxidants in maintaining human health and preventing diseases. Many studies have shown the association between the levels of ROS and the MMP. Therefore, it is an essential task for free radical scavenging to suppress ROS production. The toxicity of Cr (VI) produces oxidative damage in cells that are protected through the action of the anthocyanin-containing anti-oxidant factor. In vivo tests carried out to determine the response of the cells to Cr (VI) and LRMA in terms of cell survival rate, ROS, MMP, and autophagy-related genes showed that cell damage was alleviated in the presence of anthocyanin at various levels (Figure 1A,B). The scavenging ability of LRMA to free radicals is shown in Figure 2. LRMA had a certain ability to scavenge free radicals. With an increasing concentration, the scavenging ability significantly improved, which was similar to those of other anthocyanin species. LRMA’s ability to scavenge free radicals increased with an increasing concentration. LRMA treatment also increased the MMP (Figure 3A,B). A connection was found between LC3 and the p62’s formation of a complex in the Cr (VI) group, eventually leading to the degradation of the lysosome and indicating autophagy activation. However, LRMA treatment interrupted the combination of LC3 and the p62, suggesting autophagy inhibition (Figure 4A,D). To prove whether the PINK1/Parkin pathway was essential for LRMA-modified mitophagy, we expressed Parkin in LRMA-treated cells. The expression results are shown in Figure 5A–B. Besides promoting autophagosome LC3II expression, Cr (VI) enhances the expression of autophagy regulatory protein ATG5 and Beclin-1 (Figure 4G). Cr (VI) in cells facilitates the transfer of Parkin from the cytoplasm to the outer mitochondrial membrane and facilitates PINK1 accumulation in the outer mitochondrial membrane. When LRMA is added, Cr (VI)-induced mitochondrial damage and mitophagy in DF-1 cells could be relieved, and Parkin’s transfer from the cytoplasm to mitochondria and the recruitment of PINK1 on mitochondria could be reduced (Figure 5A–E). All in all, these data indicated that LRMA inhibited mitophagy in DF-1 cells by restraining the PINK1/Parkin pathway. Figure 6 is a graphical indication of the mechanism according to this article’s research.

## 5. Conclusions

This work explored the regulatory effect of LRMA in Cr (VI)-induced mitophagy in DF-1 cells. LRMA could reduce DF-1 cell damage, mitochondrial damage, and mitophagy caused by Cr (VI). Mechanistically, LRMA inhibits mitophagy activity by downregulating the PINK1/Parkin pathway, reducing mitochondrial damage, and conferring a protective effects on DF-1 cells.

## Data Availability

Not applicable.
